# Critical limb-threatening ischaemia and microvascular transformation: clinical implications

**DOI:** 10.1093/eurheartj/ehad562

**Published:** 2023-08-27

**Authors:** Santeri Tarvainen, Galina Wirth, Greta Juusola, Olli Hautero, Kari Kalliokoski, Tanja Sjöros, Veikko Nikulainen, Jouni Taavitsainen, Jarkko Hytönen, Crister Frimodig, Krista Happonen, Tuomas Selander, Tomi Laitinen, Harri H Hakovirta, Juhani Knuuti, Nihay Laham-Karam, Juha Hartikainen, Kimmo Mäkinen, Seppo Ylä-Herttuala, Petra Korpisalo

**Affiliations:** Heart Center, Kuopio University Hospital, Puijonlaaksontie 2, 70210 Kuopio, Finland; A.I. Virtanen Institute for Molecular Sciences, University of Eastern Finland, Kuopio, Finland; Heart Center, Kuopio University Hospital, Puijonlaaksontie 2, 70210 Kuopio, Finland; A.I. Virtanen Institute for Molecular Sciences, University of Eastern Finland, Kuopio, Finland; Heart Center, Kuopio University Hospital, Puijonlaaksontie 2, 70210 Kuopio, Finland; A.I. Virtanen Institute for Molecular Sciences, University of Eastern Finland, Kuopio, Finland; Turku University Hospital, Turku, Finland; Vaasa Central Hospital, Vaasa, Finland; Turku University Hospital, Turku, Finland; Turku PET Centre, Turku, Finland; University of Turku, Turku, Finland; Åbo Akademi University, Turku, Finland; Turku University Hospital, Turku, Finland; Turku PET Centre, Turku, Finland; University of Turku, Turku, Finland; Åbo Akademi University, Turku, Finland; Turku University Hospital, Turku, Finland; Heart Center, Kuopio University Hospital, Puijonlaaksontie 2, 70210 Kuopio, Finland; A.I. Virtanen Institute for Molecular Sciences, University of Eastern Finland, Kuopio, Finland; Heart Center, Kuopio University Hospital, Puijonlaaksontie 2, 70210 Kuopio, Finland; A.I. Virtanen Institute for Molecular Sciences, University of Eastern Finland, Kuopio, Finland; Heart Center, Kuopio University Hospital, Puijonlaaksontie 2, 70210 Kuopio, Finland; Heart Center, Kuopio University Hospital, Puijonlaaksontie 2, 70210 Kuopio, Finland; Research Services, Kuopio University Hospital, Puijonlaaksontie 2, 70210 Kuopio, Finland; Imaging Center, Kuopio University Hospital, Puijonlaaksontie 2, 70210 Kuopio, Finland; Turku University Hospital, Turku, Finland; University of Turku, Turku, Finland; Satasairaala, Pori, Finland; Turku University Hospital, Turku, Finland; Turku PET Centre, Turku, Finland; University of Turku, Turku, Finland; Åbo Akademi University, Turku, Finland; A.I. Virtanen Institute for Molecular Sciences, University of Eastern Finland, Kuopio, Finland; Heart Center, Kuopio University Hospital, Puijonlaaksontie 2, 70210 Kuopio, Finland; Heart Center, Kuopio University Hospital, Puijonlaaksontie 2, 70210 Kuopio, Finland; Heart Center, Kuopio University Hospital, Puijonlaaksontie 2, 70210 Kuopio, Finland; A.I. Virtanen Institute for Molecular Sciences, University of Eastern Finland, Kuopio, Finland; Heart Center, Kuopio University Hospital, Puijonlaaksontie 2, 70210 Kuopio, Finland; A.I. Virtanen Institute for Molecular Sciences, University of Eastern Finland, Kuopio, Finland; Turku University Hospital, Turku, Finland; Turku PET Centre, Turku, Finland

**Keywords:** Chronic limb-threatening ischaemia, Peripheral arterial disease, Angiogenesis, Capillaries, Microvascular remodelling, Ischaemia, Hypoxia, Vascular endothelial, Growth factor

## Abstract

**Background and Aims:**

Clinical management of critical limb-threatening ischaemia (CLTI) is focused on prevention and treatment of atherosclerotic arterial occlusions. The role of microvascular pathology in disease progression is still largely unspecified and more importantly not utilized for treatment. The aim of this explorative study was to characterize the role of the microvasculature in CLTI pathology.

**Methods:**

Clinical high-resolution imaging of CLTI patients (*n* = 50) and muscle samples from amputated CLTI limbs (*n* = 40) were used to describe microvascular pathology of CLTI at the level of resting muscle blood flow and microvascular structure, respectively. Furthermore, a chronic, low arterial driving pressure-simulating ischaemia model in rabbits (*n* = 24) was used together with adenoviral vascular endothelial growth factor A gene transfers to study the effect of microvascular alterations on muscle outcome.

**Results:**

Resting microvascular blood flow was not depleted but displayed decreased capillary transit time (*P* < .01) in CLTI muscles. Critical limb-threatening ischaemia muscle microvasculature also exhibited capillary enlargement (*P* < .001) and further arterialization along worsening of myofibre atrophy and detaching of capillaries from myofibres. Furthermore, CLTI-like capillary transformation was shown to worsen calf muscle force production (*P* < .05) and tissue outcome (*P* < .01) under chronic ischaemia in rabbits and in healthy, normal rabbit muscle.

**Conclusions:**

These findings depict a progressive, hypoxia-driven transformation of the microvasculature in CLTI muscles, which pathologically alters blood flow dynamics and aggravates tissue damage under low arterial driving pressure. Hypoxia-driven capillary enlargement can be highly important for CLTI outcomes and should therefore be considered in further development of diagnostics and treatment of CLTI.

## Structured Graphical Abstract

**Structured Graphical Abstract:**
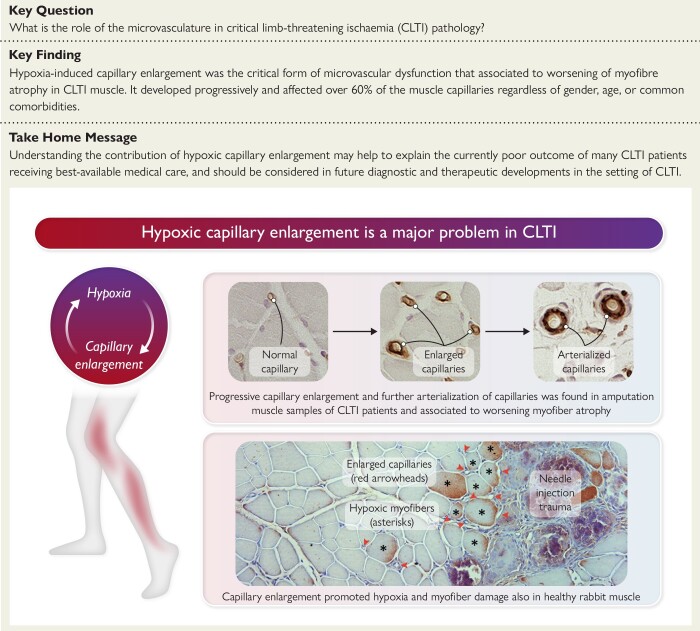
Hypoxic capillary enlargement is a major problem in chronic limb-threatening ischaemia (CLTI).

**See the editorial comment for this article ‘A conundrum of arterialized capillaries and vascular dilation in chronic limb-threatening ischaemia’, by V.C. Ganta *et al*., https://doi.org/10.1093/eurheartj/ehad826.**

## Introduction

In chronic limb-threatening ischaemia (CLTI), basic tissue metabolism at rest is compromised resulting in ischaemic rest pain, non-healing ulcers, and gangrene.^1,2^ Clinical management of CLTI is largely based on prevention and treatment of atherosclerotic arterial occlusions and yields only suboptimal results leaving patients at high risk of amputation and death.^1–3^ Beyond atherosclerotic arterial occlusions, it is known that tissue blood flow in ischaemic human muscle does not increase in response to vasodilatory stimulus.^4^ However, normal amount of resting blood flow in ischaemic human skeletal muscle has been reported in previous studies^5–8^ and presents a paradox considering CLTI with resting ischaemic symptoms.^9^ If at resting conditions severely ischaemic muscles display normal amount of muscle blood flow, it would be important to understand what actually causes the ischaemic symptoms, especially, as CLTI remains a significant, yet underappreciated, clinical problem.

On the tissue level, the muscle microvasculature is known to remodel in response to ischaemia.^10–12^ However, the contribution of ischaemic microvascular remodelling in CLTI pathology is still largely unspecified and more importantly not utilized in terms of treatment. Among ischaemic microvascular pathology, dysfunction of the vascular endothelium has been proposed to impair endothelial reactivity and angiogenesis in ischaemic muscle.^13,14^ However, clinical benefits of experimental angiogenic therapies trying to improve endothelial responsiveness have so far been limited.^1,15^ This explorative study was thus initiated to comprehensively analyse microvascular pathology of CLTI and to better understand the contribution of the microvascular changes on CLTI pathology.

## Methods

### Study design and aim

**Figure 1:**
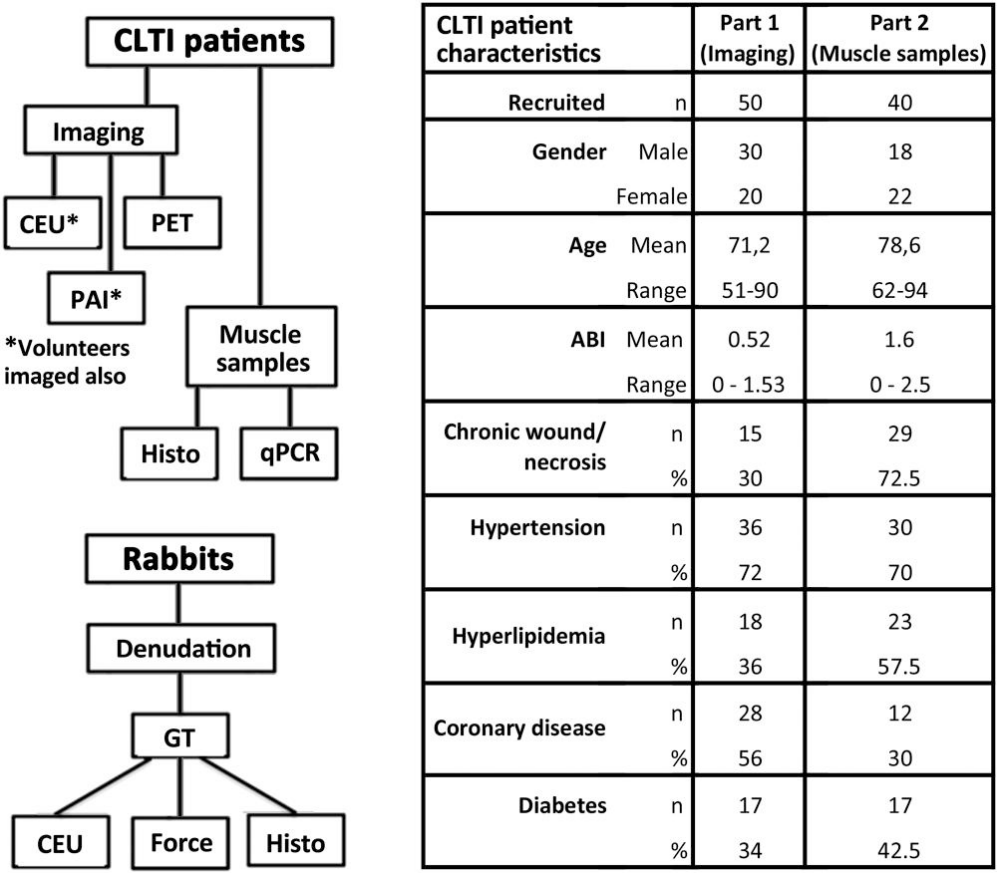
**Study design and patient characteristics.** The study is composed of three parts involving: (i) imaging of resting limb muscles in critical limb-threatening ischaemia (CLTI) patients scheduled for urgent revascularization and healthy volunteers to initially establish the paradoxical conservation of tissue blood flow under critical ischaemia, (ii) muscle sample collection from amputated CLTI limbs to characterize the structural alterations of muscle capillaries under different levels of ischaemic exposure, (iii) modelling the effects of capillary enlargement and arterialization under low arterial driving pressure in a rabbit model of chronic limb ischaemia. The characteristics of CLTI patients recruited to the study are presented in the table. CEU, contrast-enhanced ultrasound; PAI, photoacoustic imaging; PET, [^15^O]-H_2_O positron emission tomography; Histo, immunohistology; qPCR, quantitative polymerase chain reaction; Denudation, ischaemia induction via femoral artery denudation; GT, gene transfer; Force, measurement of calf muscle force production; ABI, ankle brachial index; CLTI, critical limb-threatening ischaemia

This is an explorative study characterizing the role of the microvasculature in CLTI pathology. The study applied first high-resolution imaging and muscle sampling in CLTI patients to describe the development of ischaemic microvascular changes (*Figure 1*). Subsequently, a low perfusion pressure-simulating ischaemia model in rabbits was used to study the effect of ischaemic microvascular remodelling on muscle outcome. Furthermore, the effect of capillary enlargement on myofibre histopathology was studied after adenoviral vascular endothelial growth factor A (AdVEGF-A) gene transfer to normoxic muscle.

### Subjects for clinical studies

Clinical data collection took place at Kuopio and Turku University Hospitals. Inclusion criteria for patients consisted of severe CLTI (Rutherford classification scale 4–6) and need for urgent invasive treatment [endovascular or surgical revascularization (*n* = 50) or leg amputation (*n* = 40)] due to CLTI. Exclusion criteria for imaging included as follows: acute ischaemic event, leg infection, severe heart failure (NYHA classes III–IV), unstable or acute angina pectoris, known septal defect of the heart, uncontrolled or resistant hypertension, severe chronic lung disease, pregnancy, lactation, and lack of cooperation or mental inability to give informed consent. Healthy controls for imaging (*n* = 7) were males (age range 24–30 years) without ischaemic symptoms, medications, or subjective impairment in daily exercise. Exclusion criteria for amputation patients included prior amputations (excluding minor toe amputations) and septic infections. Medical data of patients were reviewed from hospital records to confirm CLTI severity and other significant characteristics (*Figure 1*).

### Modelling of chronic ischaemia

A chronic CLTI-like ischaemia model was used in rabbits (average weight 3.5 kg, *n* = 24) to simulate gradual lowering of arterial driving pressure along multiple arterial occlusions after femoral artery denudation.^16^ Upon arterial thickening, on average 8 weeks post-operation, AdVEGF-A or beta galactosidase control (AdLacZ) gene transfers (total dose 3 × 10e11vp) were performed as 30 × 0,1-mL injections to the lower limb muscles for randomly assigned rabbits to induce capillary arterialization as previously described.^17^ Animals were then monitored at one, two, and four weeks post-gene transfer to follow the effects of capillary arterialization on ischaemic recovery.

### Contrast-enhanced ultrasound imaging

Contrast-enhanced ultrasound (CEU) was used to study microvascular blood flow with Acuson (Siemens) ultrasound and Contrast Pulse Sequencing (CPS) software in anaesthetized rabbits after electrically induced exercise stimulus^18^ and in resting human subjects with: 9L4 transducer, mechanical index 0.18, frequency 7 MHz, power 75 dB, CPS gain 0, frame rate 2 Hz, and clip length 1–3 min. Distal gastrocnemius muscles, at the side of most prominent ischaemic pain in patients, were imaged after a minimum of 30-min rest in a supine position. Imaging was started at the administration of a 2.2-mL bolus of intravenous Sonovue (Bracco) contrast agent flushed with 5 mL of NaCl (0.9%). Imaging of the contralateral leg in a corresponding position was performed at minimum 15 min later. Acquired video clips were analysed using DataPro (Noesis) software in a blinded manner. Contrast-enhanced ultrasound signal intensity was measured from muscle, arterial, and venous areas as a function of time.^19^ Maximal signal intensity (relative to both blood flow and volume) expressed as body weight adjusted values (dB/kg) as well as capillary transit time (difference between venous and arterial contrast arrival times)^19^ were derived from the data. Data distorted by artefacts rising from e.g. muscle twitching, were excluded from analyses. To verify results from CEU, photoacoustic imaging (PAI)^20,21^ and positron emission tomography (PET)^22,23^ were also used to quantitate microvascular haemoglobin content and absolute perfusion in a subset of CLTI patients (see methods from Supplementary data online).

### Measurement of muscle force production

Measurement of calf muscle force production was performed on anaesthetized rabbits. Small needle electrodes (27G) were placed on both sides of the sciatic nerve on the lateral side of the thigh to electrically stimulate contractions in the calf muscles for 6 min at 3 Hz using a voltage of 75–150 V (14A11 Electromyograph, Disa).^18^ The resultant ‘kick’ or plantar flexion force was concurrently measured using a 10 N force metre (Kyowa LVS-1KA) and maximal force extracted from force–time graphs in Excel (Microsoft).

### Muscle sample collection

Muscle samples from rabbits were collected immediately after sacrifice and perfusion fixed with 1% paraformaldehyde. From patients undergoing leg amputation due to CLTI, muscle samples were collected in the operating room from different levels to establish paired samples representing ‘ischaemic’ (distal calf or foot muscle) and most normal ‘control’ (thigh or proximal calf muscle) areas. For histological analyses, tissues were fixed with 4% paraformaldehyde-sucrose overnight in +4°C and further immersed in 15% sucrose for a minimum of 12 h prior to dehydration and embedding in paraffin. Critical limb-threatening ischaemia muscle samples for RNA extraction were directly submerged in RNA-later and snap frozen in liquid nitrogen once out of the operating room.

### Immunohistological and histopathological analyses

Muscle samples (human or rabbit) were used to evaluate the relation of microvascular alterations to the severity of ischaemic muscle damage or normal muscle histopathology. Paraffin embedded muscle sections (7 µm) were stained with haematoxylin–eosin for histopathological analysis or immunohistochemical methods as previously described.^24^ Briefly, mouse-anti-human CD31 antibody (Dako, 1:50), mouse-anti-human alpha smooth muscle actin (α-SMA, Sigma, 1:250), and mouse-anti-human vascular endothelial growth factor (VEGF, Santa Cruz, 1:100) were used to immunostain endothelial cells, vascular pericytes/smooth muscle, and endogenously expressed VEGF, respectively. Furthermore, immunostainings for hypoxia inducible factor 1α (HIF-1α, 1:50) and 2α (HIF-2α, 1:250) were performed using tyramine signal amplification (Akoya Biosciences) on AdVEGF-A transduced normoxic muscles obtained from a previous study.^25^ Images were acquired using an Olympus AX70 light microscope and further analysed in a blinded manner using AnalySIS-software (Olympus) for capillary density (number/mm^2^ muscle), mean luminal area (μm^2^),^24^ and capillary size distribution (% of capillaries with larger than average normal capillary luminal area being 19 μm^2^ in humans and 13 μm^2^ in rabbits). In the rabbit muscles, comparison of differences was done against treatment and control groups whilst taking into account the level of myofibre atrophy in 10 representative images per group. In the CLTI amputation muscle samples, capillary analyses were performed according to the level of myofibre atrophy across all samples with 20 representative images per group.

### RNA extraction and analyses

Frozen amputation muscle samples (*n* = 2 × 20) were homogenized for RNA extraction using Tri-reagent (Invitrogen) with Precellys hard tissue homogenizing CK28 lysing kit in Precellys 24 homogenizer (Bertin instrument). A total of 1 µg of total RNA was used for cDNA synthesis using DNA-free RNA kit (Ambion), random primers, and RevertAid Reverse transcriptase (Fermentas). Gene expression of hypoxia inducible factor 1 subunit alpha (HIF1A), endothelial PAS domain protein 1 (EPAS1 or HIF2A), VEGFA, and the housekeeping gene TATA-box binding protein (TBP) were quantified in ischaemic vs. control samples using real time quantitative (q)PCR (StepOne Plus) with Taqman-based assays (VEGFA, HsPT.58.1149801; EPAS1, HsPT.58.2273374; HIF-1a, HsPT.58.534274; and TBP, HS.PT.58.20792004; IDTDNA) and 2× Universal Taqman PCR mix (LifeTechnology).

### Statistical methods

Patient characteristics are presented as means with range and quantitative results as means ± SEM on dotplots or timeline graphs. Statistical analysis was performed using SPSS software (IBM). Non-parametric analyses, namely Kruskal–Wallis test followed by a Mann–Whitney *U* test for pairwise comparisons, were used for all data due to limited sample size. Two-sided *P*-value < .05 was considered statistically significant, and only biologically relevant significant values have been reported. No *P*-value adjustment for multiple comparisons was made due to the explorative nature of the study. With variable group sizes due to missing data, final analysed sample numbers are represented in each figure.

## Results

### Critical limb-threatening ischaemia muscles display decreased capillary transit time and progressive capillary enlargement and arterialization

**Figure 2:**
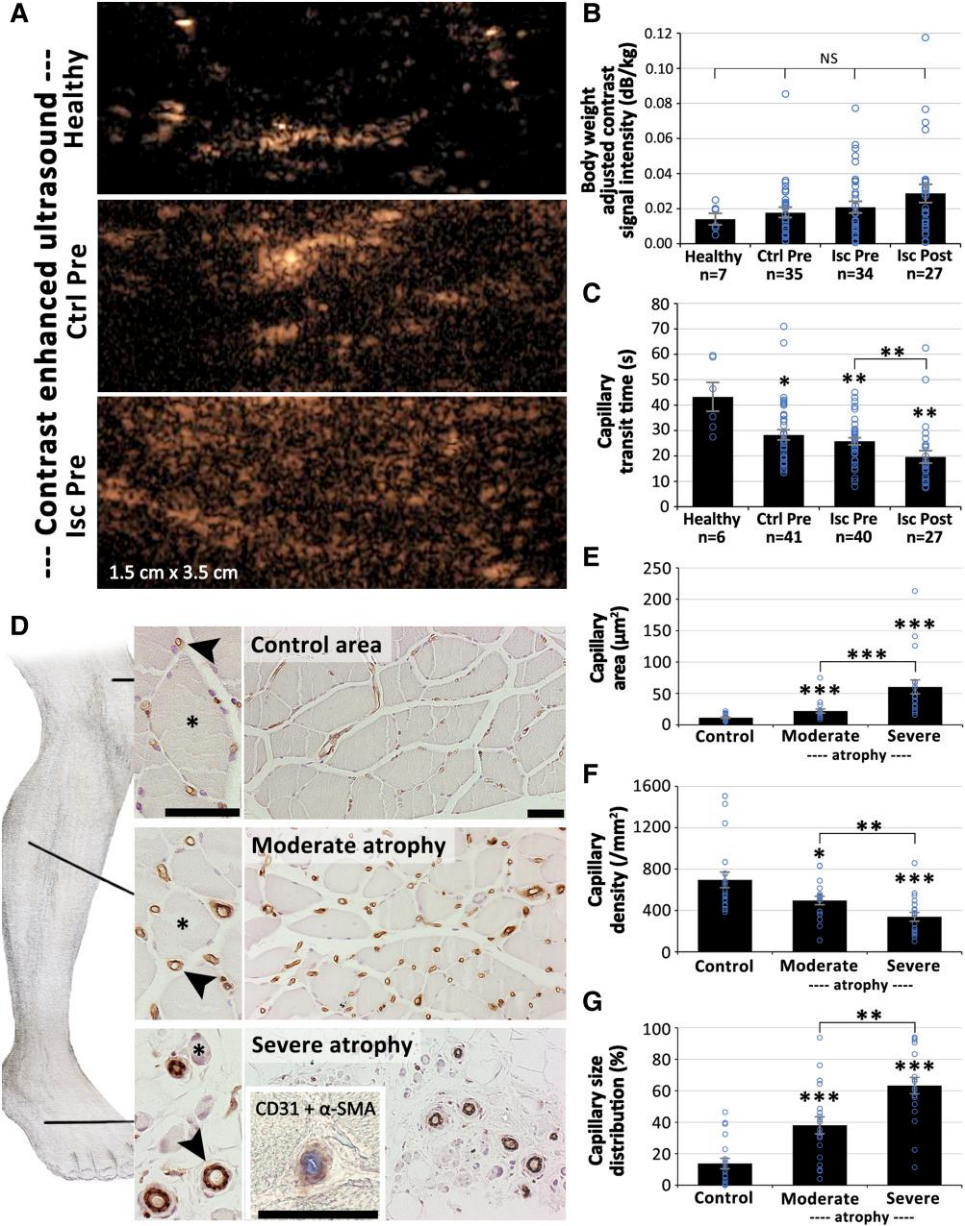
**Altered microvascular blood flow kinetics and progressive capillary arterialization in critical limb-threatening ischaemia (CLTI) muscle.** (*A*) Intravascularly delivered contrast signal (spekles) detected by ultrasound imaging was used to assess resting muscle blood flow in CLTI patients and volunteers. (*B*) Quantitation of contrast-enhanced ultrasound (CEU) signal intensity showed no significant difference in resting muscle blood flow between CLTI patients or volunteers but rather a trend towards increased signal in the ischaemic legs as compared to healthy controls. (*C*) Capillary transit time of the CEU contrast agent was significantly decreased in ischaemic muscle as compared to volunteers. (*D*) Structural transformation of muscle capillaries (arrowheads) along worsening of myofibre atrophy was seen in CD31 (brown) immunostanings. Double staining for CD31 + α-SMA (blue and brown, respectively) was used to confirm the presence of α-SMA positive pericytes on arterialized capillaries. (*E*) Increased mean capillary area, (*F*) decreased capillary density, and (*G*) increased capillary size distribution were detected along worsening of myofibre atrophy. Isc, ischaemic leg; Pre, before revascularization; Ctrl, contralateral leg of a patient; Post, after revascularization; CLTI, critical limb-threatening ischaemia; CEU, contrast-enhanced ultrasound. Scale bars 50μm. *P < .05, **P < .01, ***P < .001

Mean muscle-level microvascular blood flow, as measured with high-resolution CEU (*Figure 2A*), was not depleted even in severe CLTI requiring immediate revascularization (*Figure 2B*). The paradoxical result was confirmed also with photoacoustic- and PET imaging (see Supplementary data online, *Figure S1*). Instead, CEU displayed significantly decreased mean capillary transit time in CLTI patients as compared to healthy volunteers (*Figure 2C*, *P* < .01). Revascularization further reduced capillary transit time of the severely ischaemic legs (*Figure 2C*, *P* < .01) but did not significantly alter CEU signal intensity (*Figure 2C*). Comprehensive sampling and histopathological analysis of muscle samples from different levels of amputated CLTI limbs revealed a widespread, progressive change in capillary structure along advancement of myofibre atrophy (*Figure 2D*). Normal appearance of tiny, thin-walled capillaries (*Figure 2D*, upmost images) was rare among even thigh-level control samples in the amputated legs. Capillaries in CLTI affected muscles often displayed luminal enlargement, thickening of capillary wall and also seemed to have lost contact with adjacent atrophic myofibres (*Figure 2D*, second row images). At sites of severe myofibre atrophy, endomysial microvessels in the size range of enlarged capillaries displayed even an arterial phenotype with extremely thick walls consisting of multiple cell layers having also α-SMA positivity (*Figure 2D*, bottom images). Mean capillary area was significantly increased (*Figure 2E*, *P* < .001) and capillary density decreased (*Figure 2F*, *P* < .001) in the most atrophic muscle areas as compared to control samples. Significant capillary enlargement was consistently present across the CLTI samples regardless of co-morbidities, gender, or age distribution of the studied individuals (see Supplementary data online, *Figure S2*). Furthermore, a progressive increase in capillary size distribution, representing the fraction of capillaries affected from the total capillary pool, was detected along advancement of myofibre atrophy (*Figure 2G*, *P* < .001). In the most severely atrophic muscles, over 60% of the muscle capillaries were found enlarged (*Figure 2G*).

### Hypoxia-induced VEGF-A expression is present across the amputated critical limb-threatening ischaemia limbs

**Figure 3:**
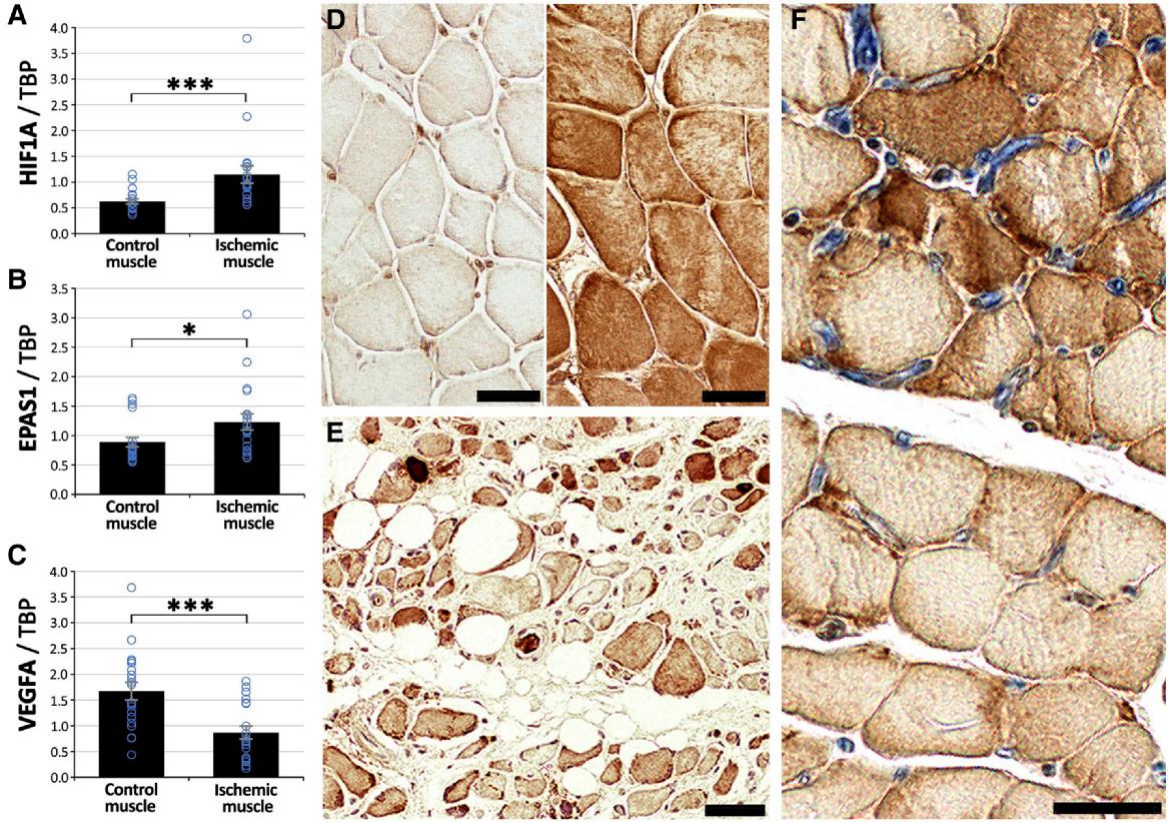
**VEGF-A immunostaining has altered pattern in ischaemic muscle and associates with capillary enlargement.** Quantification of average mRNA expressions via qPCR from amputation muscle samples displayed increased (*A*) HIF1A and (*B*) EPAS1 levels consistent with increased hypoxia but decreased (*C*) VEGFA expression in the ischaemic samples. (*D*) Amputation muscles samples from control areas showed distinct patches of VEGF-A −/+ (brown) myofibres. (*E*) Myofibres in the ischaemic amputation samples displayed heterogeneous VEGF-A expression (brown). (*F*) CD31 (blue) + VEGF-A (brown) double immunostainings display enlarged capillaries near myofibres that strongly expressed VEGF-A. Scale bars 50 μm. *P < .05

Unlike hypoxia-induced transcription factors HIF1A (*Figure 3A*, *P* < .05) and EPAS1 (*Figure 3B*, *P* < .05), mRNA levels of VEGFA were significantly decreased (*Figure 3C*, *P* < .05) in the most ischaemic samples as compared to control samples via qPCR. However, with immunohistological analyses, distinct areas could be detected with either just the microvascular endothelium positive for VEGF-A or also the myofibres strongly positive for VEGF-A (*Figure 3D*) already in the control samples, suggesting hypoxic exposure. Towards the most ischaemic samples, the pattern of VEGF-A immunostaining changed. VEGF-A immunopositivity was more heterogeneous throughout samples, and the staining intensity varied even between neighbouring myofibres in the most ischaemic samples (*Figure 3E*). Notably, even severe myofibre atrophy as such did not automatically seem to affect VEGF-A protein expression as many severely atrophied myofibre remnants were still seen strongly positive for VEGF-A (*Figure 3E*). VEGF-A was thus widely expressed throughout the amputated legs and even at the thigh-level control samples. Furthermore, double immunostaining for VEGF-A (brown) and endothelial marker CD31 (blue) in the ischaemic muscle samples demonstrated that enlarged microvascular structures were often located near myofibres with strong VEGF-A immunostaining (*Figure 3F*).

### Capillary enlargement promotes myofibre damage in rabbit skeletal muscle

**Figure 4:**
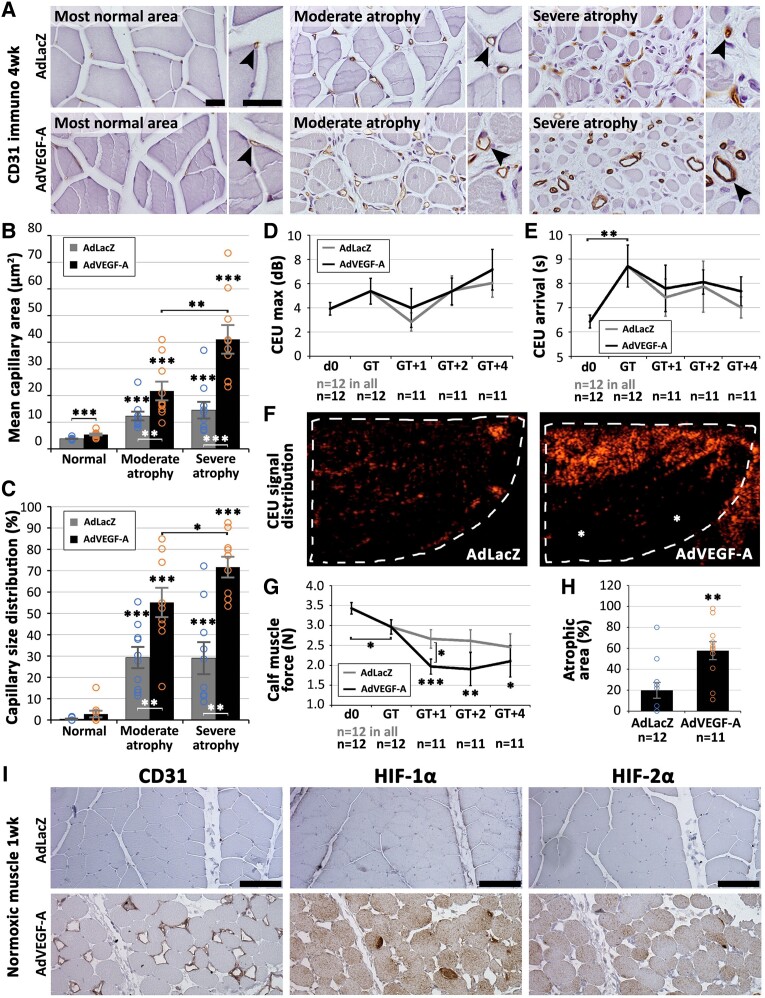
**Capillary enlargement by AdVEGF-A under low arterial driving pressure aggravates ischaemic tissue damage.** (*A*) Structural transformation of muscle capillaries (arrowheads) along worsening of myofibre atrophy seen in CD31 (brown) immunostanings 4 weeks in AdLacZ control transduced muscles and further capillary enlargement and arterialization induced by AdVEGF-A. Increased (*B*) mean capillary area and (*C*) capillary size distribution were quantified along worsening of myofibre atrophy. (*D*) Mean contrast-enhanced ultrasound (CEU) signal intensity did not significantly differ between gene transfer groups or as compared to normal (d0) values. (*E*) Mean CEU arrival time was significantly increased after the ischaemia operation but did not differ between the gene transfer groups. (*F*) Contrast-enhanced ultrasound signal (spekles) distribution in the transduced muscles (dotted line) was uneven after AdVEGF-A gene transfer leaving parts of the muscle unperfused (asterisks). (*G*) Calf muscle force production was reduced by the ischaemia operation at the time of gene transfer and further decreased after AdVEGF-A gene transfer. (*H*) Area of atrophic muscle damage increased after AdVEGF-A gene transfer. (*I*) Rounding, occasional atrophy, and increased expression of Hif-1a and Hif-2a of myofibres 1 week after AdVEGF-A gene transfer to healthy rabbit skeletal muscle as compared to changes in respective AdLacZ control. CEU, contrast-enhanced ultrasound; d0, baseline; GT, gene transfer; GT + X, X weeks after GT. Scale bar in main images 50μm, inset 25μm. *P < .05, **P < .01, ***P < .001

Similarly to CLTI muscle, capillary changes in a chronic ischaemia model in rabbits (*Figure 4A*) were associated with the severity of myofibre atrophy (*Figures 4B*). Significant capillary enlargement was detected already in the AdLacZ control transduced muscles (*Figure 4B* and *C*). Importantly, further increases in both mean capillary area (*Figure 4B*) and capillary size distribution (*Figure 4C*) were induced by AdVEGF-A gene transfer. Furthermore, similar to CLTI, microvascular blood flow was not depleted in the chronic ischaemia model at gene transfer. Blood flow did also not differ between groups during follow-up (*Figure 4D*) despite the effective capillary enlargement by AdVEGF-A. Instead, contrast arrival time was prolonged in the model at the time of gene transfer (*Figure 4E*), indicating reduced arterial driving pressure to the calf muscles. After AdVEGF-A gene transfer CEU signal distribution within the transduced muscles (dotted line in *Figure 4F*) was highly uneven leaving parts of the calf muscle void of flow (* in *Figure 3F*). Muscle force production was also impaired (*Figure 4G*, *P* < .05 at 1 week after transduction) and atrophic area increased (*Figure 4H*, *P* < .01) after AdVEGF-A gene transfer as compared to AdLacZ control. Furthermore, AdVEGF-A induced capillary enlargement in normoxic muscle was also found to result in myofibre hypoxia as indicated by myofibre positivity to both HIF-1α and HIF-2α as well as structural damage such as rounding (asterisks) of myofibres already a week after transduction (*Figure 4I*).

## Discussion

Impaired capillary function has already been associated with several disease pathologies.^26,27^ Arterialized capillaries have also been identified previously in CLTI muscle.^11,12^ This study now describes how: (i) capillary arterialization is a progressive process involving first capillary enlargement and at further stages the deposit of mural cells to surround the enlarged capillary; (ii) microvascular blood flow is not depleted, even in CLTI requiring urgent revascularization, but its kinetics are altered upon capillary enlargement; (iii) capillary enlargement is a consistent pathological feature in CLTI muscle regardless of co-morbidities, gender, or age of the studied individuals; (iv) chronic hypoxia-driven VEGF-A overexpression is a potent pathomechanistic factor initiating capillary enlargement and arterialization in CLTI muscles and worsens the outcome of chronic ischaemia also in rabbits; and (v) capillary enlargement can damage skeletal muscle myofibres under both ischaemic and normoxic conditions (*Structured Graphical Abstract*). Hereafter, we also offer an explanation to how this capillary enlargement may impair oxygen diffusion whilst leading to tissue damage. This information helps to explain how resting ischaemic symptoms can co-exist with apparently normal-in-quantity resting tissue blood flow and may offer understanding also to the currently poor outcome of some CLTI patients regardless of efforts along current medical and revascularization strategies.

Classical sprouting angiogenesis that is often considered the hallmark of VEGF-A actions during tissue development^28^ is not the typical response accompanying hypoxic VEGF-A overexpression in already vascularized CLTI muscles. These data demonstrate that microvascular remodelling associated with abundant chronic VEGF-A overexpression in CLTI muscles does not increase average capillary density but in fact promotes enlargement and eventually arterialization of pre-existing capillaries whilst significantly reducing capillary number. A similar finding after transient AdVEGF-A overexpression has already been published by us in animal models and has been considered beneficial for ischaemic recovery as it greatly improved tissue blood flow.^25^ The widespread arterialization of the capillary bed in CLTI muscle was, however, associated with aggravation of myofibre atrophy and did so also in the here reported chronic ischaemia model in rabbits whilst also impairing calf muscle force production. Explaining the differential results is potentially a critical difference in the blood flow conditions between the current study and the previously commonly used acute ischaemia models.^17,18^ Contrary to previous findings, AdVEGF-A gene transfer here in the chronic ischaemia model did not increase the overall tissue blood flow regardless of effective capillary enlargement. Instead, signs of uneven flow distribution were detected with high-resolution CEU in the AdVEGF-A transduced muscles.

**Figure 5:**
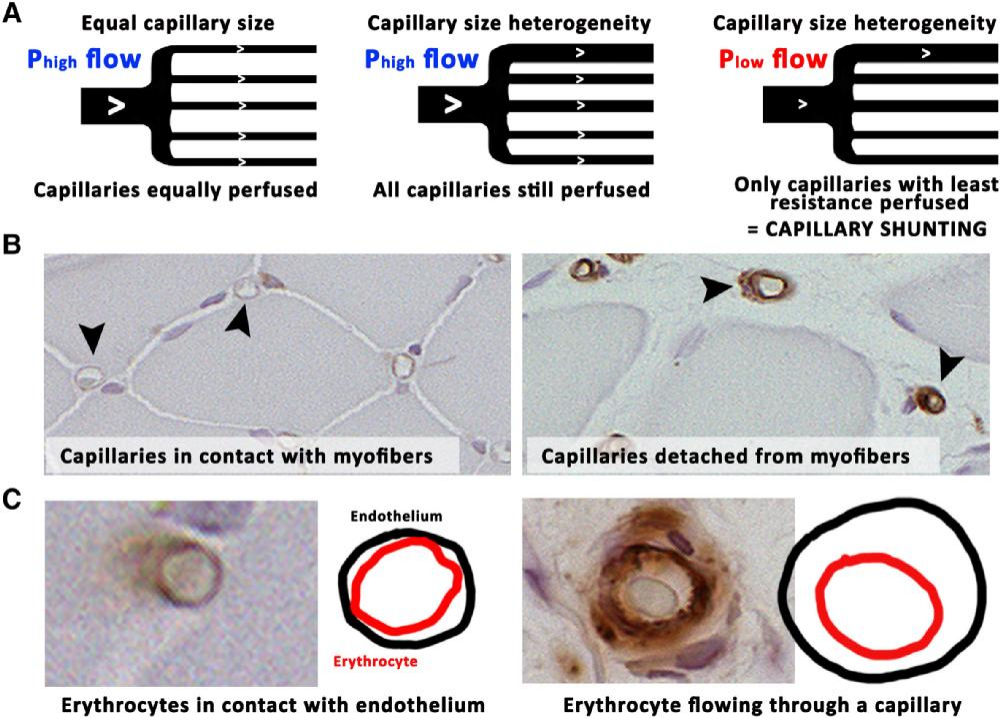
**Microvascular blood flow dynamics as well as oxygen diffusion may be compromised in critical limb-threatening ischaemia (CLTI).** (*A*) Decreased arterial driving pressure can predispose tissues with capillary size heterogeneity to shunting. Where capillaries with uniform diameter and resistance are logically equally perfused, capillary size heterogeneity alters the amount of flow in each capillary. Upon decreased arterial driving pressure, capillary size heterogeneity-mediated shunting may leave normal, high-resistance capillaries unperfused. (*B*) Capillaries normally are in thigh contact with myocytes. Capillaries in atrophic CLTI muscle, however, are often seen having lost contact with their surrounding myofibres potentially influencing oxygen diffusion.^30^ (*C*) Whilst in normal capillaries the contact surface of erythrocytes and capillary endothelial cells is maximized by squeezing of erythrocytes through the capillary tube, the enlarged capillaries may allow relatively free flow of erythrocytes decreasing contact surface. A decrease in cell membrane contacts could impair oxygen diffusion as diffusion through cell membrane phospholipids has been shown more efficient than diffusion through biological fluids.^30^ CLTI, critical limb-threatening ischaemia

Uneven flow distribution is normal for capillary networks that dynamically adapt to altered tissue needs by peripheral vasodilation and vasoconstriction.^29^ The dynamic nature of the changes can also be expected to protect tissues under physiological conditions by ensuring that no part of the microvascular network is left without perfusion for too long. Based on the current histology, prolonged hypoxia in CLTI seems, however, to have exposed the tissues to long-enduring capillary enlargement and thus can be imagined to have also distorted the normal dynamic nature of capillary flow.^10^ As flow rate is highly dependent on vessel diameter, the most enlarged capillaries under low arterial driving pressure could steal flow from smaller capillaries having higher resistance (*Figure 5A*). This capillary shunting phenomenon is supported by the significantly decreased capillary transit time detected here in CLTI muscles. As capillary endothelium is critically dependent on blood flow,^31^ long-enduring capillary shunting could also indicate a mechanism for the decreased capillary number detected here in CLTI muscles. Moreover, progressive myofibre atrophy in the presence of high-flow enduring arterialized capillaries raised further concern over the functionality of enlarged capillaries in delivering oxygen. Indeed, it has been suggested that oxygen diffusion occurs more effectively through a network of cell membrane phospholipids as opposed to delivery through biological fluids.^30^ In support of this idea, enlarged capillaries are here in CLTI muscle shown to lose contact with the adjacent myofibres (*Figure 5B*). Logically, also, the contact area of red blood cells with capillary endothelium is reduced in the enlarged capillaries (*Figure 5C*). It is thus compelling to speculate that the loss of cell membrane contacts could weaken oxygen delivery from red blood cells to the myofibre already at a very early stage of hypoxic microvascular vasodilation.

More concrete evidence of the detrimental effect of capillary enlargement on skeletal muscle myofibres was further found from re-evaluating AdVEGF-A transduced normoxic muscles from a previous study.^25^ Previously, rounding of myofibres after angiogenic gene transfers was considered a result of tissue oedema, a known side-effect of angiogenic gene therapy.^25^ Now, it is shown that these structurally damaged, initially normoxic, skeletal muscle myofibres also stain for HIF-1α and HIF-2α indicating that the rounding could in fact result from hypoxia. Capillary enlargement therefore seems to be able to damage even normoxic, healthy skeletal muscle. Notably, the type of damage caused, either in the rabbit samples or in CLTI muscle, seems to be myofibre rounding, not vast necrosis. This indicates that oxygen delivery is not completely ceased but only decreased by capillary enlargement, and there still could be potential for recovery. More studies will be needed to understand how to normalize enlarged and arterialized capillaries in CLTI muscle. Obvious value can be seen in arterial revascularization for improving arterial driving pressure of hypoxic muscle, perhaps reducing capillary shunting and re-installing effective capillary flow in the remaining normal capillaries. Also, exercise therapy that already is recognized to be highly beneficial for patients with peripheral arterial disease^1,2^ is known to alter especially capillary gene expression^32^ and therefore could exert its beneficial effects by acting on the microvasculature.

In conclusion, these data describe how hypoxic capillary enlargement, potentially via promotion of microvascular shunting and loss of cell membrane contacts, can aggravate skeletal muscle outcome. These results may help understand not only CLTI pathology but also the variable results from previous angiogenic gene therapy trials. The potential of this knowledge should be considered in terms of developing novel treatments as well as improving clinical diagnostics to recognize microvascular changes in CLTI patients. Significant benefits for patient care may well be found from normalizing ischaemic microvascular pathology.

## Supplementary Material

ehad562_Supplementary_Data

## Data Availability

Permitting agreements (concerning ownership, rights to use, immaterial property rights, or confidentiality), legislation and research ethics for third party use of the data, the datasets used and/or analysed during the current study can be made available from the corresponding author on reasonable request.
