# Supplementary material for: Critical limb-threatening ischaemia and microvascular transformation: clinical implications
Source: Eur Heart J. 2023 Aug 27;45(4):255–64. doi: 10.1093/eurheartj/ehad562 (PMC10821383; doi:10.1093/eurheartj/ehad562)
Supplement: ehad562_Supplementary_Data [file ehad562_supplementary_data.pdf]

Critical limb-threatening ischemia and microvascular transformation: clinical implications

Tarvainen S et al.

SUPPLEMENTAL DATA FILE

Absolute perfusion and hemoglobin content of CLTI muscle, and the effect of co-morbidities on the observed capillary and microvascular blood flow parameters.

Contents

1. Supplemental methods

2. Supplemental figures

3. Supplemental figure legends

4. References

1. SUPPLEMENTAL METHODS

Photoacoustic imaging

Photoacoustic imaging (PAI) combines optical spectroscopy with ultrasound imaging to yield an ultrasensitive ultrasound signal from endogenous molecules such as hemoglobin.[1] It was used here (part 1) to construct an image of the hemoglobin-filled microvascular network in lower limb muscles without any confounding effect that may be produced by using external contrast agents. A prototypic clinical PAI-device FF-PAI-CSS (Fujifilm) comprised a 3R safety class Alexandrite laser integrated into an ultrasound system giving pulsed light with energy of 80 mJ/pulse, pulse width of 50±10 ns and 10 Hz pulse repetition rate at a wavelength of 750 nm to target hemoglobin.[2] The imaging consists of a motorized 3D scan of the microvasculature with frame rate 5.0 fps and scan speed 1.0 mm/s over a 5 cm distance up to about 1,5 cm in depth with a 9 MHz central frequency linear transducer array. Similar to CEU, PAI was performed in the distal gastrocnemius muscles at rest. The average photoacoustic vascularity index representing the amount

of signal at 750 nm was calculated blindly using Fujifilm-software.[2] Furthermore, 3D reconstructions and maximum intensity projections of the reconstructions were generated using Fujifilm's Synapse 3D-viewer based software and ImageJ software, respectively. Skin layer at about 0-0.5 cm of depth was removed from the 3D stack before quantification or reconstruction.

Positron emission tomography

Positron emission tomography (PET) with $[^{15}O]$-H_2O was used to measure absolute calf muscle perfusion (ml/min/g of muscle) of 10 patients with CLTI using a D690 PET/CT scanner (GE Medical) at Turku PET Centre (part 1). Scanning area in axial (proximal-distal) direction was determined according to: a) patients own description of where the ischemic pain was more prominent or; b) lower skin temperature detected by clinical examination. After positioning and resting with the legs in the gantry for about 10 min, a CT scan of the legs was taken for attenuation correction during PET-image reconstruction. The oxygen-15 isotope and $[^{15}O]$-H_2O were produced with a Cyclone 3 cyclotron (Ion Beam Applications Inc) and a continuously working Radiowater Generator (Hidex Oy), respectively according to clinical standards.[3] Perfusion measurement was started with an intravenous injection of the $[^{15}O]$-H_2O-tracer. Continuous arterial blood sampling (5ml/min) for tracer activity was started simultaneously with the tracer infusion and continued until the end of the PET scan. PET scan was started 30s after the tracer injection and performed in 18 x 5 s, 6 x10 s, 4 x 15 s, and 5 x 30 s time frames. $[^{15}O]$-H_2O-PET imaging was performed only for patients as studies in healthy volunteers with the similar study setup had been conducted by the authors previously.[4]

Parametric perfusion images were calculated using a linearized one-tissue compartment model. Lawson-Hanson nonnegative least squares (NNLS) were used to solve general linear least squares functions. Arterial blood activity data was used for the calculation of input function. Regions of interest (ROI) were drawn manually to the anterior, posteromedial, and posterolateral muscle compartments of the leg avoiding large vessels. All the analyses were performed blinded by using Carimas 2.9 software (Turku PET Centre) to extract the mean perfusion values of the selected ROI.

2. SUPPLEMENTAL FIGURES

Supplementary Figure 1.

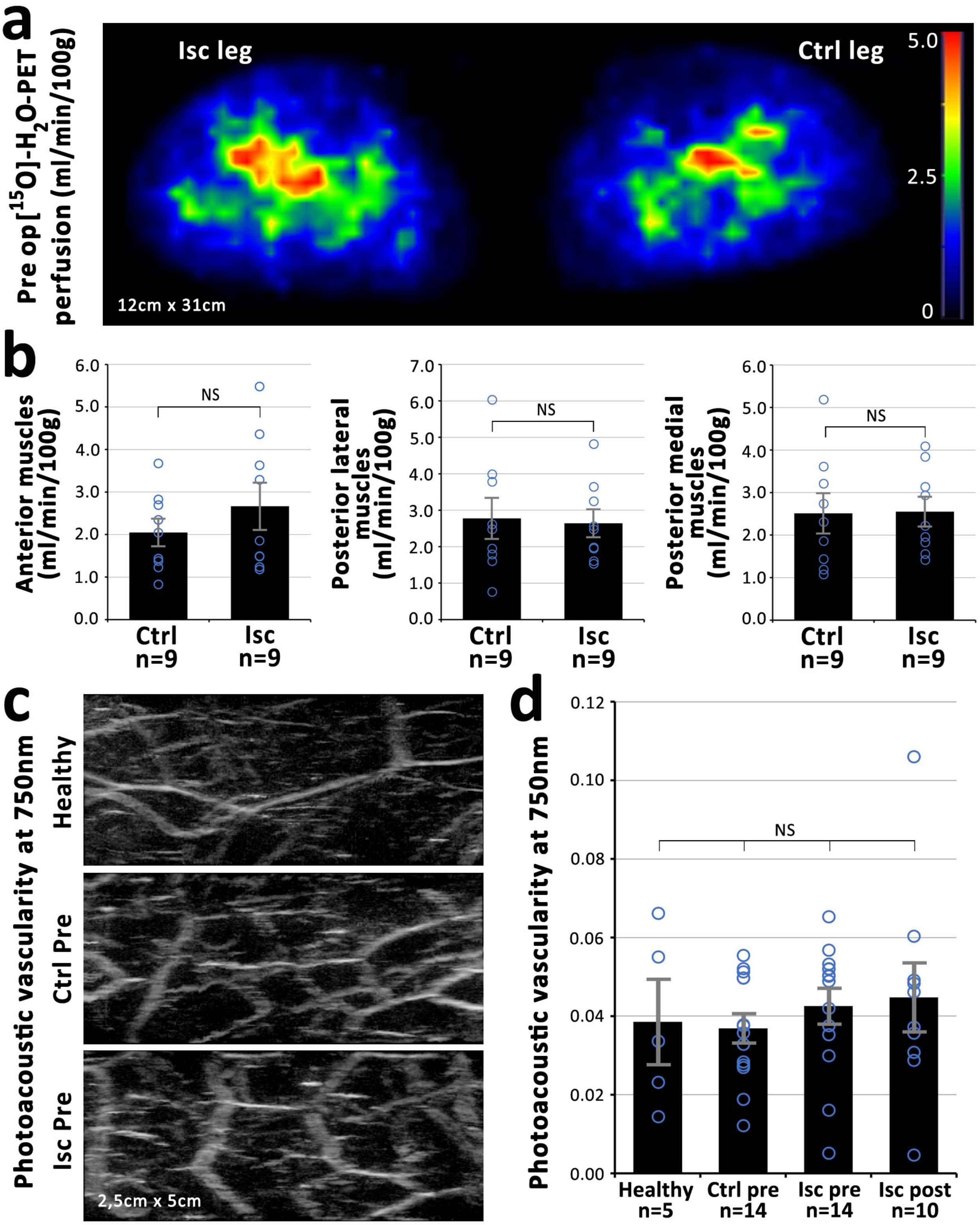

Supplementary Figure 2.

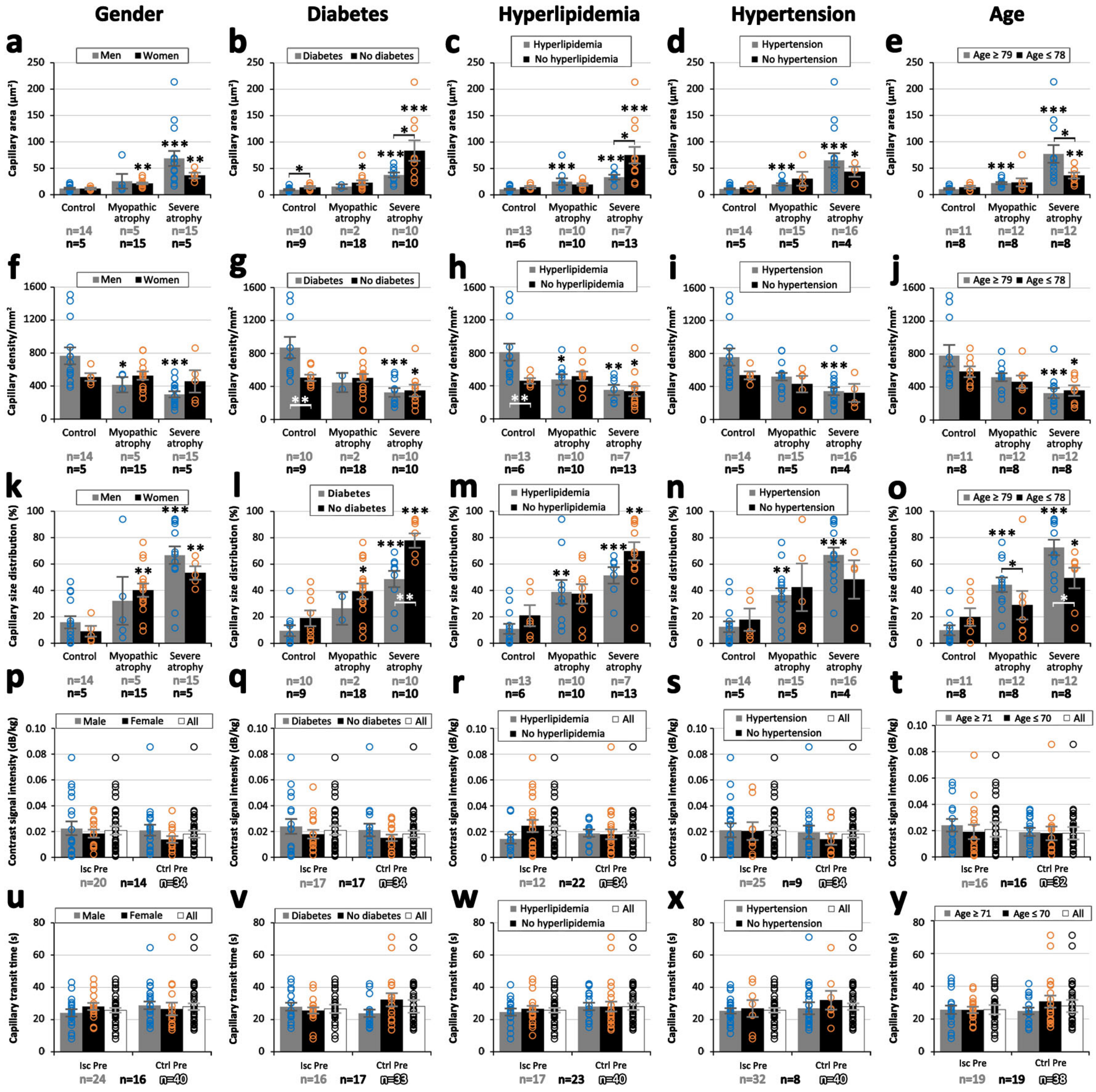

3. SUPPLEMENTAL FIGURE LEGENDS

Supplementary Figure 1. Critically ischemic CLTI legs display no decrease in absolute muscle perfusion or altered muscle hemoglobin content. a) $[^{15}O]$-H_2O-PET was used to confirm the paradoxal finding of normal resting muscle blood flow in critically ischemic legs of CLTI patients detected with CEU. **b)** Quantification of absolute muscle perfusion with $[^{15}O]$-H_2O-PET showed in average no difference in resting muscle perfusion between the critically ischemic legs of CLTI patients as compared to the contralateral non-symptomatic legs of the same patients in any of the imaged muscle compartments. The absolute muscle perfusion also ranged between 2-3ml/min/100g in all critically ischemic legs in line with previously published values from healthy individuals.[5-8] **c)** To control also for any possible changes in tissue hematocrit in the ischemic muscles, completely non-invasive photoacoustic imaging was used to construct 3D projection maps of microvascular hemoglobin. **d)** Average intensity of hemoglobin signal showed no significant difference between the critically ischemic legs of patients as compared to the controls. Lack of perfusion or decreased tissue level hematocrit therefore seem not to explain resting ischemic symptoms in even critically ischemic legs of CLTI patients.

Supplementary Figure 2. Findings related to microvascular remodeling and blood flow are consistent regardless of co-morbidities, gender or age. The possible effects of gender, diabetes, hyperlipidemia, hypertension and age on **a-e)** capillary area, **f-j)** capillary density, **k-o)** capillary size distribution, **p-t)** CEU signal intensity and **u-y)** capillary transit time we analyzed in the study population. Despite gender, co-morbidities or age distribution of the study subjects, capillary enlargement (a-e) is consistently present in CLTI muscle. The presence of diabetes (b) or hyperlipidemia (c) results in less capillary enlargement than respective controls without these diseases. However, significant capillary enlargement is still present with or without diabetes or hyperlipidemia. Also, old age (>79 years) increases the amount (e) and especially the extent (o) of capillary enlargement suggesting progressiveness of capillary remodeling. Gender, co-morbidities or age had no effect on microvascular blood flow parameters (p-y).

4. REFERENCES

1. Wang X, Pang Y, Ku G, Xie X, Stoica G, Wang LV. Noninvasive laser-induced photoacoustic tomography for structural and functional in vivo imaging of the brain. Nat Biotechnol 2003;21:803–806. doi: https://doi.org/10.1038/nbt839
2. Irisawa K, Hirota K, Hashimoto A, Murakoshi D, Ishii H, Tada T, et al. Photoacoustic imaging system for peripheral small-vessel imaging based on clinical ultrasound technology. In: Oraevsky AA, Wang L v., eds. Proc. of SPIE Vol 9708. 2016:970807. doi: https://doi.org/10.1117/12.2211352
3. Sipilä HT, Clark JC, Peltola O, Teräs M. An automatic [15O]H2O production system for heart and brain studies. J Labelled Comp Radiopharm 2001;44:S1066–S1068. doi: https://doi.org/10.1002/jlcr.25804401381
4. Heinonen I, Wendelin-Saarenhovi M, Kaskinoro K, Knuuti J, Scheinin M, Kalliokoski KK. Inhibition of α-adrenergic tone disturbs the distribution of blood flow in the exercising human limb. American Journal of Physiology-Heart and Circulatory Physiology 2013;305:H163–H172. doi: https://doi.org/10.1152/ajpheart.00925.2012
5. Heinonen I, Saltin B, Kemppainen J, Sipilä HT, Oikonen V, Nuutila P, et al. Skeletal muscle blood flow and oxygen uptake at rest and during exercise in humans: a pet study with nitric oxide and cyclooxygenase inhibition. American Journal of Physiology-Heart and Circulatory Physiology 2011;300:H1510–H1517. doi: https://doi.org/10.1152/ajpheart.00996.2010
6. Heinonen I, Nesterov SV, Kemppainen J, Nuutila P, Knuuti J, Laitio R, et al. Role of adenosine in regulating the heterogeneity of skeletal muscle blood flow during exercise in humans. J Appl Physiol (1985) 2007;103:2042–2048. doi: https://doi.org/10.1152/JAPPLPHYSIOL.00567.2007
7. Kalliokoski KK, Oikonen V, Takala TO, Sipilä H, Knuuti J, Nuutila P. Enhanced oxygen extraction and reduced flow heterogeneity in exercising muscle in endurance-trained men. Am J Physiol Endocrinol Metab 2001;280. doi: https://doi.org/10.1152/AJPENDO.2001.280.6.E1015

8. Kalliokoski KK, Kemppainen J, Larmola K, Takala TO, Peltoniemi P, Oksanen A, et al. Muscle blood flow and flow heterogeneity during exercise studied with positron emission tomography in humans. Eur J Appl Physiol 2000;83:395–401. doi: https://doi.org/10.1007/S004210000267
